# Assessment of neutrophil-to-lymphocyte ratio and platelet-to-lymphocyte ratio in patients with high myopia

**DOI:** 10.1186/s12886-022-02688-1

**Published:** 2022-11-30

**Authors:** Xin Wang, Qing He, Xiaoyu Zhao, Haoru Li, Lin Liu, Di Wu, Ruihua Wei

**Affiliations:** 1 Tianjin Key Laboratory of Retinal Functions and Diseases, Tianjin Branch of the National Clinical Research Centre for Ocular Disease, Eye Institute and School of Optometry, Tianjin Medical University Eye Hospital, No. 251, Fukang Road, Nankai District, Tianjin, 300384 China; 2 Beijing Yanqing District Hospital (Peking University Third Hospital Yanqing Hospital), No. 28, East Shuncheng Street, Yanqing District, Beijing, China

**Keywords:** High myopia, Neutrophil-to-lymphocyte ratio, Platelet-to-lymphocyte ratio, Systemic inflammation

## Abstract

**Background:**

Previous reports have suggested that inflammation levels play a crucial role in the pathogenesis of high myopia (HM). This study aimed to investigate the relationship between HM and systemic inflammation using the neutrophil-to-lymphocyte ratio (NLR) and platelet-to-lymphocyte ratio (PLR).

**Methods:**

Overall, 100 age- and sex-matched participants were recruited for the study, including 50 participants each in the non-HM (NHM) and HM groups. Ocular examinations and blood tests were performed. The NLR and PLR values were calculated from complete blood counts. Receiver operating characteristic (ROC) curves and optimal cut-off values were used to determine the optimal values of the NLR and PLR to distinguish between the HM and NHM groups.

**Results:**

The values of NLR and PLR were significantly elevated in the HM group compared with those in the NHM group (*P* < 0.001 and *P* = 0.010, respectively). Axial length (AL) was significantly correlated with the NLR (*r* = 0.367, *P* < 0.001) and PLR (*r* = 0.262, *P* = 0.009). In the ROC analysis, the NLR value to distinguish between the HM and NHM groups was 0.728; the best cut-off value was 2.68, with 76% sensitivity and 62% specificity. The PLR value to distinguish between the HM and NHM groups was 0.650; the best cut-off value was 139.69, with 52% sensitivity and 76% specificity.

**Conclusion:**

The findings of this study indicate that the development of HM may be associated with systemic inflammation measured using the NLR and PLR.

**Trial registration:**

The study was registered on December 28, 2021 (http://www.chictr.org.cn; ChiCTR2100054834).

## Background

Myopia is a global public health concern. The global prevalence of myopia is estimated to increase by 50% and that of high myopia (HM) by 10% in 2050 [1]. HM can lead to macular degeneration and is a common cause of vision loss in adults [2]. The prevalence of myopia is significantly increasing in East Asia [3].

The current generally accepted definition of high myopia is refraction lesser than − 6.00 diopters or an eye axial length (AL) greater than 26 mm [4, 5]. The pathogenesis of HM is unclear, and a correlation between HM and inflammation has been proposed [6]. A study found that the expression levels of interleukin 6 (IL-6) and matrix metalloproteinase-2 (MMP-2) in the aqueous humour (AH) were significantly higher in patients with HM than in those with non-highly myopic eyes, and the expression of IL-6 and MMP-2 were significantly correlated with AL [7]. In addition, γ-glutamyltyrosine and 12-oxo-20-trihydroxy-leukotriene B4, which are two metabolites associated with inflammation, were identified as two significant metabolic predictors of HM [8]. Thus, the results of these previous studies suggest that inflammation levels play a crucial role in the pathogenesis of HM.

The correlation between inflammatory cytokines in AH and HM has already been studied, and inflammatory cytokines in AH are more strongly correlated with myopic severity than those in the plasma [9, 10]. Recently, the neutrophil-to-lymphocyte ratio (NLR) and platelet-to-lymphocyte ratio (PLR) have been used as new inflammatory markers to evaluate the severity of inflammation. Because these ratios can be obtained easily and quickly, an increasing number of studies have focused on these indicators. Significant changes in the NLR and PLR have prognostic value in several common acute and chronic diseases, including tumors [11, 12], coronary artery disease [13], stroke [14], and rheumatic diseases [15].

As inflammation plays a crucial role in the pathological processes of HM, this study aimed to investigate the NLR and PLR in patients with HM and non-high myopia (NHM) to determine whether they could be potential biomarkers in patients with HM. To date, few studies have reported differences in the NLR and PLR between patients with HM and NHM.

## Methods

This study included 100 eyes from 100 participants. This study was approved by the Medical Ethics Committee of the Tianjin Medical University Eye Hospital, and the procedures adhered to the tenets of the Declaration of Helsinki. Informed consent was obtained from all included participants. The study was registered on December 28, 2021 (http://www.chictr.org.cn; ChiCTR2100054834).

The participants were recruited from Tianjin Medical University Eye Hospital in China between January and March 2022. After propensity-score matching for age and sex, 50 patients were included in the NHM (Group I) and HM (Group II) groups, respectively. All participants were aged ≥40 years. The AL was < 26 mm in Group I and ≥ 26 mm in Group II. Participants with ocular or systemic diseases, including hypertension, diabetes, coronary artery disease, tumors, thyroid disease, immune disorders, glaucoma, age-related macular degeneration (AMD), ocular hypertension, corneal dilatation diseases such as keratoconus, fundal disease other than HM, or ocular trauma that could affect the NLR and PLR were excluded from this study. Pregnant women were also excluded.

A slit-lamp examination, intraocular pressure examination, and examination of best-corrected visual acuity were performed on all recruited participants. Photographs of the fundus were obtained using a CR-2 camera (Canon Medical Systems, Otawara, Japan), and swept-source optical coherence tomography/optical coherence tomography angiography (VG200S; SVision Imaging, Henan, China) images were used to examine the retinal fundus. In addition, an IOL Master 700 (Zeiss, Jena, Germany) examination was performed to measure the AL.

For all participants, 3–5 mL venous blood samples were collected from the antecubital vein in the early morning and mixed with an anti-coagulant (EDTA-K2). Complete blood count (CBC) measurements were performed within 2 h using an automated blood cell analyzer (XT-1800i; Sysmex, Kobe, Japan), and the levels of neutrophils, lymphocytes, and platelets were measured as part of the automated CBC. The NLR and PLR were calculated. Notably, the NLR and PLR algorithms included the ratio of neutrophils to lymphocytes and platelets to lymphocytes, respectively.

### Statistical analysis

Statistical analyses were performed using SPSS software (version 26.0; IBM, Armonk, NY). The normality of each continuous variable was tested using the Kolmogorov–Smirnov normality test. Continuous variables with a normal distribution were expressed as means ± standard deviations, while continuous variables without a normal distribution were expressed as medians and interquartile ranges. An independent t-test, Mann–Whitney U test, and χ^2^ test were used to compare the continuous and categorical variables, as appropriate. Spearman’s correlation was used to analyze the correlations between age, AL, and NLR or PLR. The area under the receiver operating characteristic (ROC) curve (AUC), specificity, sensitivity, and cut-off values were used to determine the accuracy of the NLR and PLR for distinguishing between HM and NHM. Statistical significance was set at *P* < 0.05.

## Results

There were 50 patients each in Group I and Group II, with mean ages of 63.10 ± 8.31 (43–82) and 63.86 ± 7.61 (51–79) years, respectively. There were no significant differences in age or sex between the two groups (*P* > 0.05). The AL of Group II was significantly longer than that of Group I (29.57 ± 2.30 vs. 23.46 ± 0.90, *P* < 0.001), as shown in Table 1. There were significant differences in NLR and PLR between the two groups (Table 2; *P* < 0.05).

**Table 1:** General characteristics of Group I and Group II

	Group I (*n* = 50)	Group II (*n* = 50)	t	*P*
Age	63.10 ± 8.31	63.86 ± 7.61	−0.477	0.634
Sex			0.694^a^	0.405
Male	20 (55.56%)	16 (44.44%)		
Female	30 (46.88%)	34 (53.12%)		
AL	23.46 ± 0.90	29.57 ± 2.30	−8.617^b^	**< 0.001**
IOP	14.61 ± 3.20	14.54 ± 3.31	−0.107 ^b^	0.915

*AL* axial length

significant *p* values (*P* < 0.05) are in bold

^a^χ2 test

^b^Mann–Whitney U test

**Table 2:** Comparison of laboratory parameters between Group I and Group II

	Group I (*n* = 50)	Group II (*n* = 50)	Z	*P*
Neutrophil	3.36 ± 0.96	3.80 ± 1.09	−1.889	0.059
Lymphocyte	2.14 ± 0.77	1.75 ± 0.55	−2.444	**0.015**
Monocyte	0.44 ± 0.14	0.44 ± 0.16	−0.400	0.689
Platelet	232.92 ± 52.06	234.06 ± 53.86	−0.090	0.929
NLR	1.68 ± 0.55	2.38 ± 1.12	−3.926	**< 0.001**
PLR	118.05 ± 35.78	143.16 ± 47.75	−2.578	**0.010**
MPV	9.88 ± 0.93	9.59 ± 0.70	−1.298	0.194
PDW	11.22 ± 1.90	10.92 ± 1.58	−0.562	0.574

*NLR* neutrophil-to-lymphocyte ratio, *PLR* platelet-to-lymphocyte ratio, *MPV* mean platelet volume, *PDW* platelet distribution width

significant *p* values (*P* < 0.05) are in bold

**Fig. 1:**
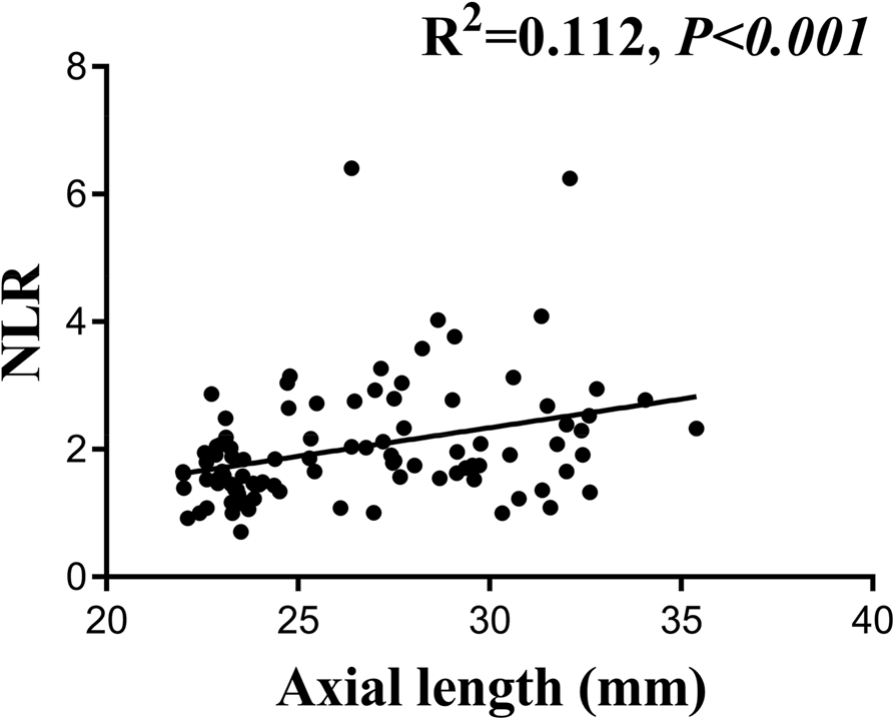
Association between axial length and neutrophil-to-lymphocyte ratio (NLR)

**Fig. 2:**
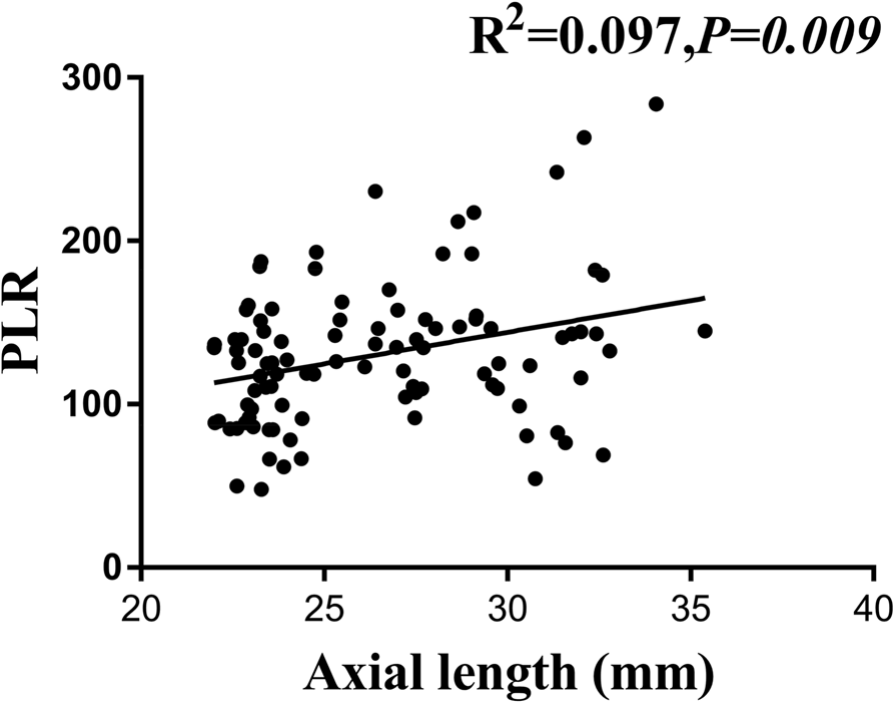
Association between axial length and platelet-to-lymphocyte ratio (PLR)

**Fig. 3:**
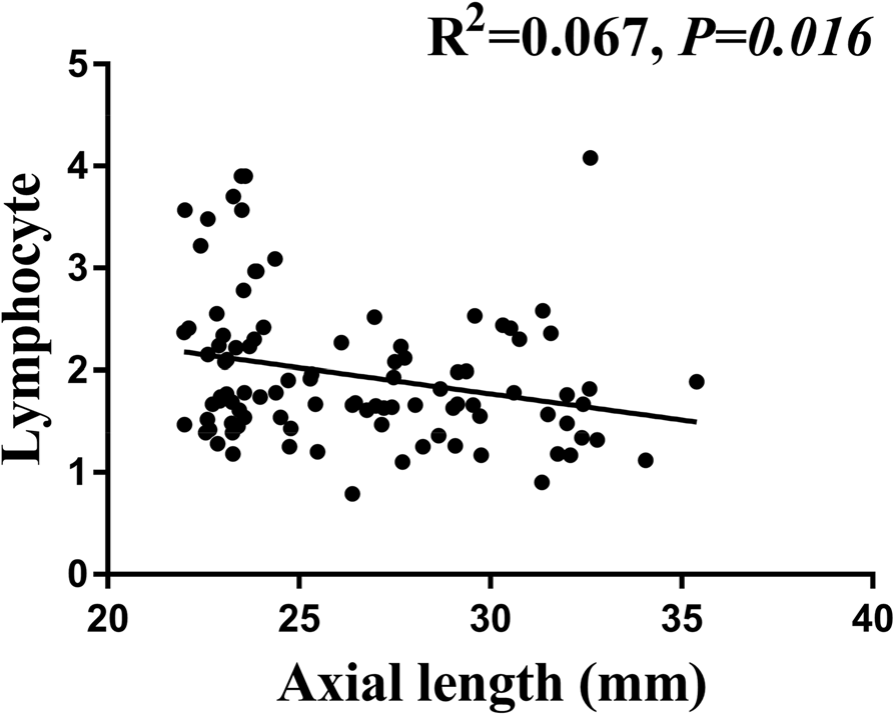
Association between axial length and lymphocyte

The correlation analysis showed no correlation between age and NLR (*P* = 0.307) or PLR (*P* = 0.647). There was no correlation between IOP and NLR (*P* = 0.904) or PLR (*P* = 0.433). AL was significantly correlated with the NLR (*r* = 0.367, *P* < 0.001) (Fig. 1), PLR (*r* = 0.262, *P* = 0.009) (Fig. 2), and Lymphocyte (*r* = − 0.240, *P* = 0.016) (Fig. 3).

**Fig. 4:**
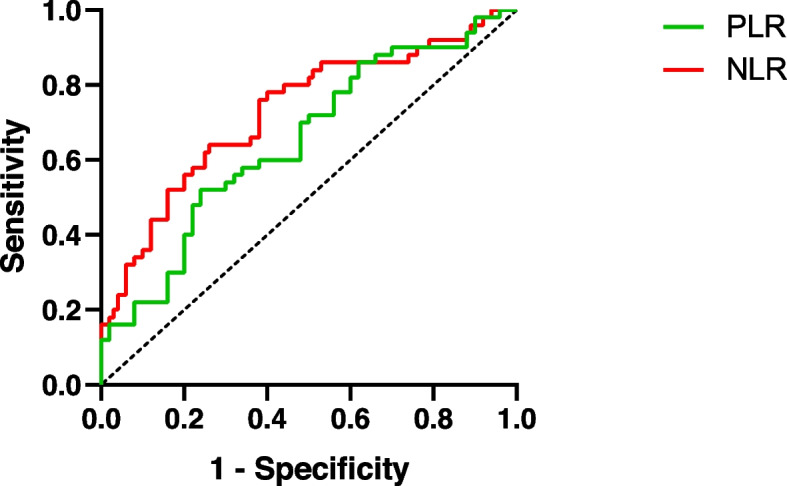
Receiver operating characteristic (ROC) curve analysis to diagnose high myopia. NLR, neutrophil-to-lymphocyte ratio; PLR, platelet-to-lymphocyte ratio

ROC analysis was used to distinguish between the two groups. The AUC of the NLR was 0.728 (standard error, 0.051; *P* < 0.001). The best cut-off point was 2.68, and the Youden index was 0.38, with a sensitivity of 76% and a specificity of 62%. The AUC of the PLR was 0.650 (standard error, 0.055; *P* = 0.010). The best cut-off point was 139.69, and the Youden index was 0.28, with a sensitivity of 52% and a specificity of 76% (Fig. 4).

## Discussion

In the current study, we compared the differences in NLR and PLR between patients with HM and NHM. Our findings revealed that the NLR and PLR were significantly higher in the HM group than in the NHM group. In addition, AL was positively correlated with the NLR and PLR. Our study suggests that the NLR and PLR are new indicators of inflammation associated with HM.

Myopia is a significant public health problem, and the prevalence of myopia has increased over time [1]. The development of myopia is considered to be determined by a combination of genetic and environmental factors [16, 17], although the exact pathogenesis and mechanism of progression of HM remain unclear. Several studies have demonstrated a correlation between inflammation and myopia [18, 19]. Systemic inflammatory diseases, including type 1 diabetes mellitus, systemic lupus erythematosus, and Vogt–Koyanagi–Harada disease, as well as inflammatory eye diseases such as multifocal choroiditis, punctuate inner choroidopathy, sclerochoroidal inflammation, and juvenile idiopathic arthritis-related uveitis are correlated with the occurrence of myopia [18, 20]. Experimental and clinical studies have reported that the development of myopia is related to the degradation of the scleral extracellular matrix and scleral remodeling [21, 22]. A longitudinal study conducted over 26 years that investigated changes in the refractive error of patients with juvenile chronic arthritis (JCA) showed that the incidence of myopia in patients with JCA was higher than that in control participants, which may be related to the effects of chronic inflammation on scleral connective tissue [23].

Clinical and experimental studies have further elucidated the role of inflammation in the pathogenesis of myopia [20, 24, 25]. Lin et al. [20] reported that compared with non-monocular form-deprivation animals, the levels of transcription factors, including nuclear factor-kappa B (NF-κB) and c-Fos and inflammatory cytokines such as IL-6, tumor necrosis factor (TNF-α), transforming growth factor (TGF-β), and interleukin 1β (IL-1β) were higher in the retina and sclera of monocular form-deprivation animals. Anti-inflammatory drugs can slow the progression of myopia. IL-6, TNF-α, and TGF-β activate the transcription factor NF-κB, and MMP2 is a target of NF-κB [20, 26]. The upregulation of MMP2 expression promotes the development of myopia via the cleaving of scleral collagen, degradation of the extracellular matrix, and reduction of scleral biomechanics [27, 28]. Additionally, the levels of IL-6, MMP-2, and TGF-β in the AH of highly myopic eyes were found to be higher than those in non-highly myopic eyes [9, 10, 29]. The results of these previous studies reveal that the level of intraocular inflammation may be an important pathological mechanism of HM.

Studies have confirmed that the development of HM is associated with oxidative stress and as the AL increases, the retinal pigment epithelium and choroid become atrophied. Moreover, oxidative stress occurs in a hypoxic environment [30]. Noma et al. [31] demonstrated that hypoxia and oxidative stress stimulate vascular endothelial cells to produce IL-6 and interleukin 8 (IL-8). IL-8 is an important chemokine that activates neutrophil infiltration in the retina. Excessive production of reactive oxygen species is known to disrupt the balance between antioxidant defense and free radical production, which plays an important role in the pathogenesis of many eye diseases [32], including myopia [33].

Information on systemic inflammation can be obtained using a variety of biochemical and hematological markers; however, most of them are time-consuming and expensive [34]. In contrast, the NLR and PLR are relatively easy to obtain and are widely considered biological markers of systemic inflammation [35]. In addition, many ophthalmic diseases are associated with the NLR and PLR, including keratoconus [36], retinal vein occlusion [37], diabetic retinopathy [38], primary angle-closure glaucoma [39], AMD [40], and thyroid-associated ophthalmopathy [41]. Relatively few studies have compared the correlation of HM with the NLR and PLR. Erel et al. [42] first reported a correlation of axial HM with the NLR and PLR; they concluded in their study that NLR and PLR were higher in HM with non-retinopathy and HM with retinopathy groups than in the control group. Additionally, they reported no difference between HM with non-retinopathy and HM with retinopathy groups; however, the HM and NHM groups in their study were not matched for age. In the current study, after propensity-score matching for age and sex, we found that the NLR and PLR were significantly higher in the HM group than in the NHM group. Furthermore, we evaluated the correlation of AL with the NLR and PLR and observed a positive correlation (*r* = 0.367, 0.262; *P* < 0.001, *P* = 0.009, respectively). Karaca et al. [36] hypothesized that myopia activates oxidative stress response and that the human retina is susceptible to oxidative stress. Sequentially, oxidative stress can activate inflammatory responses, and local inflammation can increase inflammatory responses in the blood [8]. This mechanism may explain the elevation of the NLR and PLR, which are inflammatory biomarkers, in the HM group in this study; however, longitudinal and animal studies are needed to confirm these results.

In our study, the AUCs of the NLR and PLR were 0.728 and 0.650, respectively, indicating that the NLR and PLR have diagnostic potential for axial HM. It has also been suggested that HM is correlated with systemic inflammation. In this study, a cut-off NLR to distinguish between the HM and NHM groups was 2.68, with a sensitivity of 76% and a specificity of 62%. The cut-off PLR was 139.69, with a sensitivity of 52% and specificity of 76%. The similar AUC values for the NLR and PLR further suggested their usefulness as biomarkers to identify patients with HM.

This study has some limitations. First, the sample size was relatively small. Second, correlations between systemic and ocular inflammation levels in patients with HM could not be assessed. Finally, although we tried our best to exclude the effect of systemic and local inflammation on the NLR and PLR, routine testing may not have ruled out all systemic inflammatory conditions and may have impacted our findings.

## Conclusions

In conclusion, the NLR and PLR, which are indicators of systemic inflammatory properties, were significantly elevated in the HM group, suggesting that myopia development may be associated with systemic inflammation. Changes in ocular and systemic inflammatory factors are important topics of future research regarding the pathogenesis of myopia.

## Data Availability

The datasets used and/or analyzed during the current study are available from the corresponding author upon reasonable request.

## Abbreviations

AH: Aqueous humour
AL: Axial length
AMD: Age-related macular degeneration
AUC: Area under the curve
HM: High myopia
IL-1β: Interleukin 1β
IL-6: Interleukin 6
IL-8: Interleukin 8
JCA: Juvenile chronic arthritis
MMP-2: Matrix metalloproteinase-2
NHM: Non-high myopia
NF-κB: Nuclear factor-kappa B
NLR: Neutrophil-to-lymphocyte ratio
PLR: Platelet-to-lymphocyte ratio
ROC: Receiver operating characteristic
TGF-β: Transforming growth factor
TNF-α: Tumor necrosis factor
